# Supplementary material for: Content-rich biological network constructed by mining PubMed abstracts
Source: BMC Bioinformatics. 2004 Oct 8;5:147. doi: 10.1186/1471-2105-5-147 (PMC528731; doi:10.1186/1471-2105-5-147)
Supplement: Additional File 2 — The original results of the above study (non-essential files are deleted to keep the file size under the limit set by BMC bioinformatics). [file 1471-2105-5-147-S2.bz2 › chilibotAdditionalFile2/dip20/405ID10681548E10065/html/IL6_OSM.html]

 


 **IL6** and **OSM** 
  
Found 446 abstracts in PubMed, retrieved 20.  
 

 What does Google say? 
 PDF only 
| .edu only 

---

**Interactive relationship** (e.g. stimulation, inhibition, etc)

**Neutral relationship**- Oncostatin M  [ **OSM** ]  belongs to the interleukin 6  [ **IL6** ]  family of cytokines and acts as a multifunctional cytokine during murine embryogenesis and in inflammatory reactions.  Ref: 12814362 Eur J Neurosci, 2003
- Oncostatin M  [ **OSM** ]   **OSM**  is a multifucntinal cytokine that belongs to the IL 6  [ **IL6** ]  family.  Ref: 12855584 Blood< MedlineTA>Blood, 2003
- Oncostatin M  [ **OSM** ]   **OSM**  is a multifunctional cytokine that belongs to the Interleukin IL 6  [ **IL6** ]  subfamily.  Ref: 12811586 Rev Physiol Biochem Pharmacol< MedlineTA>Rev Physiol Biochem Pharmacol, 2003
- Oncostatin M  [ **OSM** ]   **OSM**  is a member of the interleukin 6  [ **IL6** ]  cytokine family, which is involved in definitive hematopoiesis, the development of liver, and local inflammation.  Ref: 12831858 Neuroscience, 2003
- Compared to other members in the IL 6  [ **IL6** ]  subfamily cytokines, M17 s cysteine positions and gene organisation are similar to those of oncostatin M  [ **OSM** ]  and LIF, although amino acid identities are only 15 17%.  Ref: 12543126 Dev Comp Immunol, 2003
- CONCLUSION IL 1, but not IL 6  [ **IL6** ] , TNFalpha, or iNOS, plays an important role in joint disease induced by intraarticular gene transfer of  **OSM**  in mice.  Ref: 12794844 Arthritis Rheum, 2003
- METHODS An adenoviral vector expressing murine  **OSM**  was injected into the joints of naive wild type mice and mice deficient for interleukin 1 IL 1, IL 6  [ **IL6** ] , tumor necrosis factor alpha TNFalpha, or inducible nitric oxide synthase iNOS.  Ref: 12794844 Arthritis Rheum, 2003
- IL 6  [ **IL6** ]  type cytokines exert their action via the signal transducers gp130, LIF receptor and  **OSM**  receptor leading to the activation of the JAK STAT and MAPK cascades.This review focuses on recent progress in the understanding of the molecular mechanisms of IL 6  [ **IL6** ]  type cytokine signal transduction.  Ref: 12773095 Biochem J< MedlineTA>Biochem J, 2003
- We conclude that a AZA inhibits the release of  **OSM** , IL 6  [ **IL6** ] , and IL 11 exclusively in RA diseased MNC, b Patient s MNC release subnormal amounts of LIF,  **OSM** , and IL 11, and c RA derived monocytes probably down regulate  **OSM**  release by phytohemagglutinin activated MNC.  Ref: 12543079 Cytokine, 2002

**Non-interactive relationship** (e.g. studied together, co-existance, homology, etc.)

- Effect of azacytidine in the release of leukemia inhibitory factor, oncostatin m  [ **OSM** ] , interleukin IL 6  [ **IL6** ] , and IL 11 by mononuclear cells of patients with refractory anemia.  Ref: 12543079 Cytokine, 2002
- We studied the connection between the secretion of TNF alpha, IL 6  [ **IL6** ] ,  **OSM**  and NO by hamster peritoneal macrophages animals bearing two forms of hamster transplantable melanomas of common origin but differing in growth rates, levels of differentiation and immunogenicity and the cytotoxic effect of these macrophages on the cells of those two melanoma lines.  Ref: 12659693 Int J Immunopathol Pharmacol< MedlineTA>Int J Immunopathol Pharmacol, 2002
- We show here that hepatocyte growth factor HGF  [ **IL6** ]  induced early transition of albumin ALB negative stem cells to ALB positive hepatic precursors resembling hepatoblasts and then oncostatin M  [ **OSM** ]   **OSM**  promoted their differentiation to tryptophan dioxygenase TO positive mature hepatocytes.  Ref: 12702664 Development< MedlineTA>Development, 2002
- Oncostatin M  [ **OSM** ]   **OSM** , a cytokine of the interleukin 6  [ **IL6** ]  family, is expressed in rheumatoid arthritis, multiple sclerosis, multiple myeloma, and other inflammatory and neoplastic conditions.  Ref: 12730964 Glia, 2003
- Studies with cultured cells and mouse models of disease have ascribed proinflammatory and anti inflammatory functions to oncostatin M  [ **OSM** ]   **OSM**  and the related cytokine, interleukin 6  [ **IL6** ]  IL 6  [ **IL6** ] .  Ref: 12856331 J Interferon Cytokine Res, 2003
- Hepatocyte specific gene expression in BMCs was identified by immunocytochemistry and reverse transcription polymerase chain reaction.RESULTS Bone marrow cell derived hepatocyte like colonies appeared after several days of coculture in medium containing HSLF, oncostatin M  [ **OSM** ]   **OSM**  and hepatocyte growth factor HGF  [ **IL6** ] .  Ref: 12821039 J Hepatol, 2003
- Adult mice were challenged with IL 6  [ **IL6** ] , oncostatin M  [ **OSM** ]   **OSM** , and LPS.  Ref: 12891556 Gastroenterology, 2003
- AZA down regulated  **OSM** , IL 6  [ **IL6** ] , and IL 11 release by MNC of patients but not by MNC from healthy individuals.  Ref: 12543079 Cytokine, 2002
- Molecular cloning and characterisation of a carp Cyprinus carpio cytokine like cDNA that shares sequence similarity with IL 6  [ **IL6** ]  subfamily cytokines CNTF,  **OSM**  and LIF.  Ref: 12543126 Dev Comp Immunol, 2003
- Transdifferentiation was enhanced by 5 azacytidine treatment, and by HSLF,  **OSM**  and HGF  [ **IL6** ] .  Ref: 12821039 J Hepatol, 2003
- Leukemia inhibitory factor LIF, oncostatin M  [ **OSM** ]   **OSM** , interleukin IL 6  [ **IL6** ] , and IL 11 regulate hemopoiesis and LIF,  **OSM** , and IL 6  [ **IL6** ]  also inhibit the proliferation of myeloid leukemic cell lines through the signal transducing subunit gp130.  Ref: 12543079 Cytokine, 2002
- Oncostatin M  [ **OSM** ]   **OSM**  is an interleukin IL 6  [ **IL6** ]  family cytokine that we have previously shown can synergize with a number of proinflammatory cytokines to promote the release of collagen from cartilage in explant culture.  Ref: 12759253 Am J Pathol, 2003
- The IL 6  [ **IL6** ]  family cytokine oncostatin M  [ **OSM** ]  was also capable of activating MAPK, STAT3, STAT1, Akt, and p38 in both WT and SYF cells.  Ref: 12764151 J Biol Chem< MedlineTA>J Biol Chem, 2003
- after IL 6  [ **IL6** ]  and  **OSM**  stimulation, gp130 dependent pathways STAT3, APR gene expression were completely blocked in the liver of these animals.  Ref: 12891556 Gastroenterology, 2003
- Here, we examined the effect of systemic administration of adenoviral Ad vectors encoding either murine  **OSM**  AdMuOSM or murine IL 6  [ **IL6** ]  AdMuIL 6 in a mouse model of colitis.  Ref: 12856331 J Interferon Cytokine Res, 2003
- These include preproglucagon, APOJ, cadherin2, phosphoglucoisomerase, oncostatin M  [ **OSM** ] , PAX6, HGF  [ **IL6** ] , and cytokeratin 18.  Ref: 12606813 Mol Med, 2002
- Following the induction of liver injury with acetaminophen APAP, a time dependent increase in liver mRNA expression of IL 6  [ **IL6** ]  and its family members IL 11, leukemia inhibitory factor, and oncostatin M  [ **OSM** ]  was observed in wild type WT mice, suggesting a possible hepatoprotective role played by this cytokine family.  Ref: 12705907 Biochem Biophys Res Commun, 2003
- The IL 6  [ **IL6** ]  type cytokines IL 6  [ **IL6** ] , IL 11, LIF,  **OSM** , CNTF, CT 1 and CLC are an important family of mediators involved in the regulation of the acute phase response to injury and infection.  Ref: 12773095 Biochem J< MedlineTA>Biochem J, 2003
